# Burden of Cardiovascular diseases attributable to risk factors in Brazil: data from the "Global Burden of Disease 2019" study

**DOI:** 10.1590/0037-8682-0263-2021

**Published:** 2022-01-28

**Authors:** Luisa Campos Caldeira Brant, Bruno Ramos Nascimento, Guilherme Augusto Veloso, Crizian Saar Gomes, Carisi Polanczyk, Gláucia Maria Moraes de Oliveira, Luisa Sorio Flor, Emmanuela Gakidou, Antonio Luiz Pinho Ribeiro, Deborah Carvalho Malta

**Affiliations:** 1 Universidade Federal de Minas Gerais, Faculdade de Medicina e Hospital das Clínicas, Belo Horizonte, MG, Brasil.; 2 Universidade Federal de Minas Gerais, Departamento de Estatística, Programa de Pós-Graduação em Estatística, Belo Horizonte, MG, Brasil.; 3 Universidade Federal de Minas Gerais, Faculdade de Medicina, Departamento de Medicina Preventiva e Social, Programa de Pós-Graduação em Saúde Pública, Belo Horizonte, MG, Brasil.; 4 Universidade Federal do Rio Grande do Sul, Faculdade de Medicina, Instituto de Avaliação de Tecnologia em Saúde, Porto Alegre, RS, Brasil.; 5 Universidade Federal do Rio de Janeiro, Rio de Janeiro, RJ, Brasil.; 6 University of Washington, Institute of Health Metrics and Evaluation, Seattle, USA.; 7 Universidade Federal de Minas Gerais, Escola de Enfermagem, Departamento de Enfermagem Materno-Infantil e Saúde Pública, Belo Horizonte, MG, Brasil.

**Keywords:** Cardiovascular disease, Risk factors, Population attributable risk, Global Burden of Disease, Brazil.

## Abstract

**INTRODUCTION:**

To better understand trends in the main cause of death in Brazil, we sought to analyze the burden of cardiovascular risk factors (RF) and cardiovascular diseases (CVD) attributable to specific RFs in Brazil from 1990 to 2019, using the estimates from the GBD 2019 study.

**METHODS:**

To estimate RF exposure, the Summary Exposure Value (SEV) was used, whereas for disease burden attributed to RF, mortality and disability-adjusted life-years (DALY) due to CVD were used. For comparisons over time and between states, we compared age-standardized rates. The sociodemographic index (SDI) was used as a marker of socioeconomic conditions.

**RESULTS:**

In 2019, 83% of CVD mortality in Brazil was attributable to RF. For SEV, there was a reduction in smoking and environmental RF, but an increase in metabolic RF. High systolic blood pressure and dietary risks continue to be the main RF for CVD mortality and DALY. While there was a decline in age-standardized mortality rates attributable to the evaluated RF, there was also a stability or increase in crude mortality rates, with the exception of smoking. It is important to highlight the increase in the risk of death attributable to a high body mass index. Regarding the analysis per state, SEVs and mortality attributable to RF were higher in those states with lower SDIs.

**CONCLUSIONS:**

Despite the reduction in CVD mortality and DALY rates attributable to RF, the stability or increase in crude rates attributable to metabolic RFs is worrisome, requiring investments and a renewal of health policies.

## INTRODUCTION

Cardiovascular diseases (CVDss) are the main cause of health loss in Brazil and worldwide, and are also responsible for high health costs^1^ ^,^ ^2^ ^.^ Among the CVDss, responsible for 27.3% of the deaths in Brazil in 2017, ischemic heart disease (IHD) and stroke are the main causes of death, contributing respectively with 32.1% and 28.2% of the deaths caused by CVDs in 2019^3^. Although the national age-standardized CVDs mortality rate was in decline between 1990 and 2017 (342 and 178 per 100,000 inhabitants, respectively), the number of deaths caused by CVDs increased during the period (266,958 and 388,268, respectively) as a result of population growth and aging^3^. Moreover, the burden of CVDs is unequal among Brazilian states, which was higher in those with lower socioeconomic development. Therefore, there is a need for specific policies which address the problem according to location^4^.

Social, environmental, behavioral, and metabolic risk factors (RF) are determining factors for the loss of health due to CVDs^1^. A large portion of the burden of CVDs can be attributed to modifiable RF, such as blood pressure, levels of glycemia, and high LDL, which are, in turn, associated with other behavioral RF CVDs, like inadequate diet and the lack of physical activity^1^ ^,^ ^5^. Therefore, it is essential to understand the trends of RF for CVDs to direct public policies that address the problem and emphasize health promotion. The Global Burden of Disease (GBD) study is a multinational collaboration that provides comparable and consistent information about the health of populations over time. In 2019, the risk factor estimates were revised and updated^6^.

The aim of this study is to analyze the trends of cardiovascular RF as well as the burden of CVDss attributable to those specific RF in Brazil from 1990 to 2019, using the estimates from the GBD 2019 study. 

## METHODS

This study used the estimates of cardiovascular RF provided by the GBD 2019 study, conducted by the Institute of Health Metrics and Evaluation (IHME), available in http://ghdx.healthdata.org/^7^.

**FIGURE 1:**
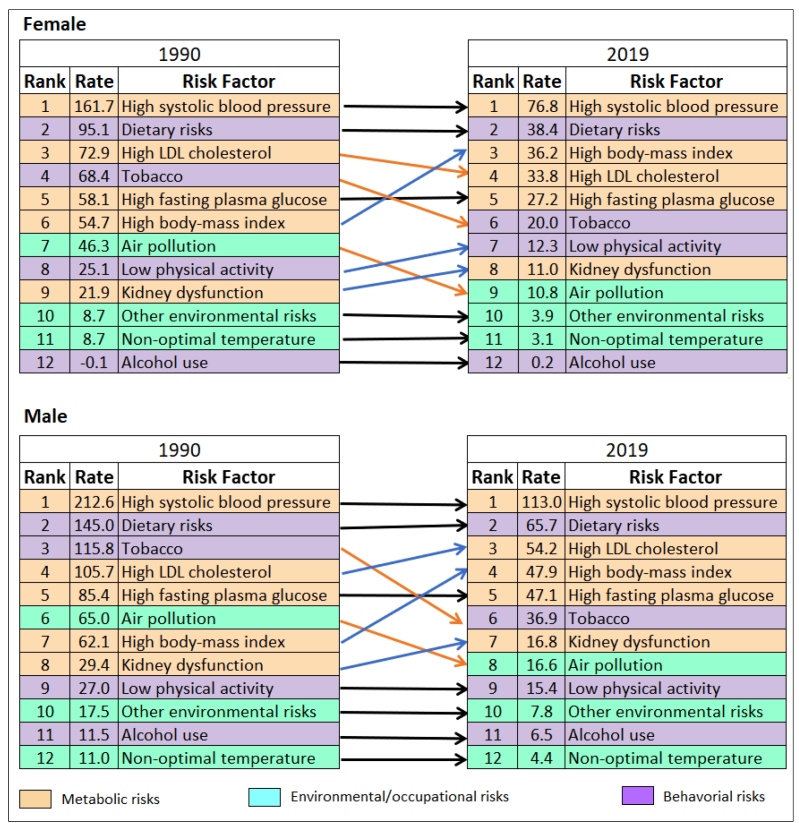
Ranking of age-standardized mortality rates (per 100,000 inhabitants) for cardiovascular diseases attributable to risk factors in 1990 and 2019 in Brazil, for females and males.

The GBD uses a hierarchical list of RF, which are analyzed in 4 levels. Level 1 divides the RF in 3 groups: metabolic, behavioral, and environmental. Level 2 details the risk factors from level 1, including 20 risk factors. Levels 3 and 4 go further in this detailing. Altogether, in 2019, the GBD study analyzed 87 RF^6^. The current study analyzed hierarchic levels 1 and 2, in which 12 RF were evaluated, as listed in Figure 1.

For Brazil, over 200 data sources were consulted, including the existing national inquiries, such as the National Health Survey (NHS), the Surveillance of RF for disease protection through telephone inquiry (VIGITEL, Portuguese), the National Household Sample Survey (PNAD, in Portuguese), the National Student Survey (NSE), and cohort studies^8^ ^-^ ^13^. 

To calculate the burden attributable to RF, the GBD followed the structure established for comparative risk assessment (CRA). The CRA follows 5 main steps: 1) to estimate mean levels of exposure to each RF for each age-sex-location-year combination, using available sources, such as home inquiries, administrative data, census data, vital records, and environmental measures. Once data is identified, different definitions are adjusted to a standardized exposure definitions, and adjustments for sex and standardized age groups are also applied if necessary. Finally, data are modelled using either spatiotemporal Gaussian process regression or DisMod-MR 2.1, which combine data from different sources and generate final exposure estimates with uncertainty intervals (95% UI) for each year, location, sex, and age group^14^; 2) To identify risk-outcome pairs according to available literature evidence; 3) To synthesize relative risks (RR) identified in published cohort or case-control studies through meta-analysis and meta-regression methods. The RR used in this study can assume a continuous or categorical format depending on the evidence available for each outcome. RR are the same for morbidity and mortality, and are applied to males and females, as well as for every country and geographic region; 4) To estimate the Theoretical Minimum Risk Exposure Level (TMREL), defined as the minimum level of exposure to each RF, in which the probability of the occurrence of a given event is the least likely. The TMREL is used to calculate the population attributable factor (PAF) for different causes of death, diseases, or disabilities; and 5) To calculate the PAF, defined as the proportion of risk that would be reduced in a given year if the exposure to a risk in the past was reduced to an ideal exposure scenario^6^ ^,^ ^12^. Methods for estimating PAFs are described in detail elsewhere^6^.

As an example, the TMREL for eleven RF evaluated in this study are: 1) Systolic blood pressure (SBP): 110 to 115 mm Hg; 2) Fasting plasma glucose: 85 to 99 mg/dL; 3) Cholesterol LDL: between 27 and 50 mg/dL ; 4) Body mass index (BMI): 20 to 25 kg/m^2^ for adults; 5) Kidney function: albumin creatinine ratio < 30 mg/g or glomerular filtration rate > 60 mL/min by 1.73 m^2^; 6) environmental air pollution: 2.4 to 5.9 μg/m3; 7) tobacco: no exposure, including passive smoking; 8) dietary risks, daily consumption of 1 to 5 g of salt and 200 to 400 g of fruits and vegetables, among others; 9) physical activity: 8,000 METs daily; 10) alcohol: no consumption; 11) ideal temperature: 25.6ºC^6^.

To evaluate exposure to RF, the GBD uses the Summary Exposure Value (SEV), which represents the prevalence weighted by the risk. The SEV scale ranges from 0 to 100%, 0% representing no exposure to the risk and 100% representing the maximum exposure. A decline in SEV means reduced exposure, while an increase means the opposite. Further details about the calculation of the SEVs have been published elsewhere^6^ ^,^ ^12^.

Definitions for CVDs have been standardized^1^. Ischemic heart disease (IHD) encompasses acute myocardial infarction^15^, stable angina (defined by the Rose Angina Questionnaire), chronic IHD, and cardiac insufficiency (CI) due to IHD. For strokes, this study considered acute signs of cerebral dysfunction, which last > 24h or cause death. Peripheral arterial disease of the lower limbs was defined by the ankle-brachial index < 0.9, whereas for aorta aneurysm, this study considered thoracic and abdominal aneurysms. Atrial fibrillation (AF) and flutter were diagnosed by EKG. For hypertensive cardiac disease (HCD), symptomatic heart failure (HF) was considered because of the direct and indirect effects of hypertension over the long term. Myocardiopathy was defined as symptomatic HF due to primary myocardial disease, or its exposure to toxins, and acute myocarditis was defined as an acute and self-limited condition caused by inflammation. For endocarditis and rheumatic cardiac disease, clinical diagnosis was used, and estimates for rheumatic cardiac disease included cases identified by clinical history, physical exam, or echocardiographic criteria standardized for a definitive disease. For non-rheumatic valve diseases, this study considered calcific diseases of the aortic valve, degenerative mitral valve disease, among others^1^ ^,^ ^7^.

In this study, the measures used to describe the burden of CVDss attributable to RF were mortality and disability-adjusted life years (DALYs) for Brazil and its states, from 1990 to 2019. 

In the GBD, mortality for Brazil was estimated using data from the Mortality Information System (SIM, in Portuguese), codified by the international classification of disease (ICD)^16^. To correct problems in the quality of information on causes of death, corrections were made for the under-reporting of deaths and for causes considered of little use to public health, called *garbage codes*. Redistribution algorithms for the garbage codes were developed by the GBD study, taking into consideration evidence from various sources, such as medical literature, expert opinions and statistical techniques^17^. 

The calculation of DALYs is the sum of the years of life lost (YLL) due to premature death, maintaining as a reference the maximum life expectancy observed globally, with the Years Lived with Disability (YLD). YLD represent the burden of non-fatal diseases and are determined by the prevalence of the condition multiplied by the disability weight caused by that condition. The prevalence of each condition was estimated through data representative of the populations, including cohort studies, registries, population inquiries, and administrative data, with the use of statistical methods for adjustments of different definitions and methods. Disability weights reflect the severity of different conditions and were developed through interviews with the general public^7^.

For comparisons over time and between Brazilian states, age-standardized rates computed with the age composition in 2019 were used^18^. For the other analyses, non-standardized rates are shown. A ranking of the RF contributing to the CVDs burden was created to highlight the changes that occurred between 1990 and 2019, according to sex, as well as the differences among the 27 Brazilian states in 2019. The 95% UI were calculated and are presented for each estimate, as described previously^1^.

The percentage of change in the mortality rate attributable to selected RF between 1990 and 2019 was analyzed according to the Socio-Demographic Index (SDI), a composite measurement of per capita income, level of education, and total fertility rate, which allows for comparison between locations in terms of their level of development. The SDI ranges from 0 (least developed) to 1 (most developed)^19^.

Statistical analysis was conducted in RStudio (RStudio Team, 2019) and figures were produced using the ggplot2 package.

The GBD-Brazil project was approved by the Research Ethics Committee of the Universidade Federal de Minas Gerais (UFMG), logged under protocol number CAAE - 62803316.7.0000.5149. 

## RESULTS

CVDss were responsible for 28.2% (95% UI 26.0%-29.4%) of deaths in Brazil in 2019, 82.6% (95% UI 80.0%-85.2%) of which were attributable to the RF assessed by the GBD ( Supplementary Material Figure 1).  Supplementary Material Table 1 shows SEV for each cardiovascular RF, by sex and year, and the percentage of change between 1990 and 2019. Risks can be divided into 3 groups: reduction in exposure, increase in exposure, or non-significant changes. For both sexes, a decline was observed for air pollution (-59.9.0%; UI95%: -73.6 - -40.0), other environmental risks (residential radon, lead exposure) (-38.3%; UI95%:-47.9 - -27.1), and tobacco (-33.7%; UI95%:-38.2 - -28.2). As for those with an increase, high BMI (+110.2%; UI95%:78.6 - 161.7), alcohol use (+41%; UI95%:24.2 - 63.4), glycemia (+15.1%; UI95%: 9.3 - 21.2), high LDL cholesterol (+11.8%; UI95%: 6.9 - 17.2), and kidney dysfunction (+12%; UI95%: 8.4 - 17.2) stand out. For SBP, dietary risks, lack of physical activity, and non-ideal temperature, the change was minimal, despite the high SEV. 

Figure 1 shows the ranking of age-standardized CVDs mortality rates attributable to RF, for males and females, in 1990 and 2019. A reduction was observed in the rates attributable to all RF for both sexes. High SBP and dietary risks remained as the two main RF for mortality by CVDs for both sexes in 2019. Over time, high BMI moved from 6^th^ to 3^rd^ place for women and from 7^th^ to 4^th^ place for men, whereas kidney dysfunction went from 9^th^ to 8^th^ place for women and from 8^th^ to 7^th^ place for men. The lack of physical activity remained as the 9^th^ leading risk factor for CVDs mortality for men, but moved from 8^th^ to 7^th^ place for women. High LDL cholesterol moved down for women and up for men between 1990 and 2019. Tobacco and air pollution moved to lower positions for both men and for women.  Supplementary Material Figure 2 shows a similar pattern for the DALY rates attributable to risk factors. 

**FIGURE 2:**
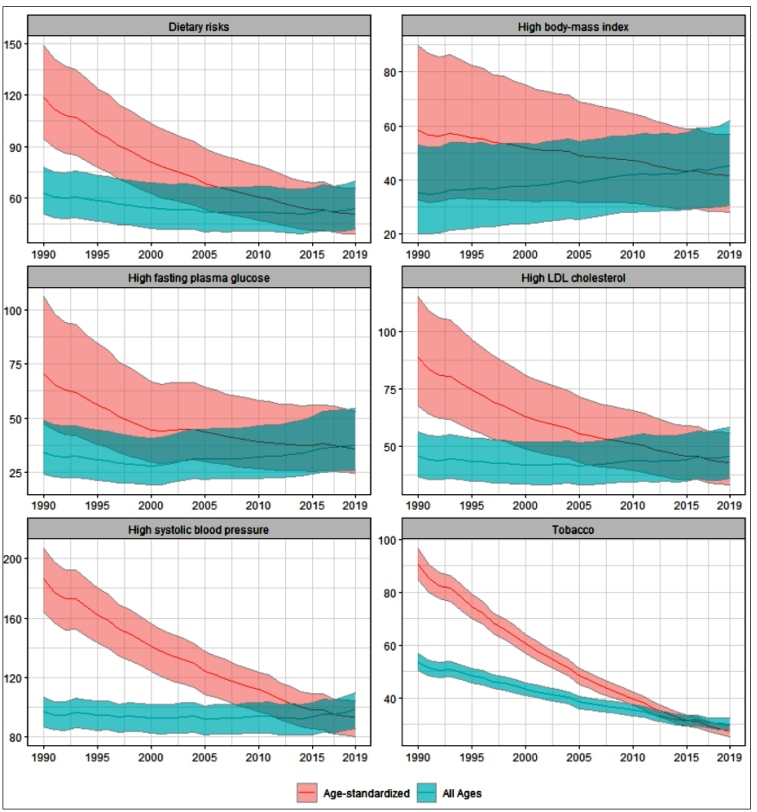
Time trends for crude and age-standardized mortality rates (per 100,000 inhabitants) for cardiovascular diseases attributable to selected risk factors with 95% uncertainty intervals, for both sexes. Brazil, 1990 to 2019.

Supplementary Material Table 2 shows the all-age and age-standardized CVDs mortality rates attributable to RF. An increase was observed in the all-age mortality rates attributable to high BMI, kidney dysfunction, and low physical activity, while tobacco, air pollution, dietary risks and other environmental showed a reduction. The other RF showed a relatively stable trend, considering the UIs. The largest relative increase was observed for high BMI, going from 35 (95% UI, 20-53) in 1990 to 46 (95% UI, 31-62) per 100,000 inhabitants in 2019 ( Supplementary Material Table 2). By contrast, when the age-standardized rates are analyzed, a decrease can be observed for all the RF, especially for tobacco, high SBP, high plasma glucose, high LDL-cholesterol, and dietary risks. Figure 2 shows these trends for selected SF. 

**FIGURE 3:**
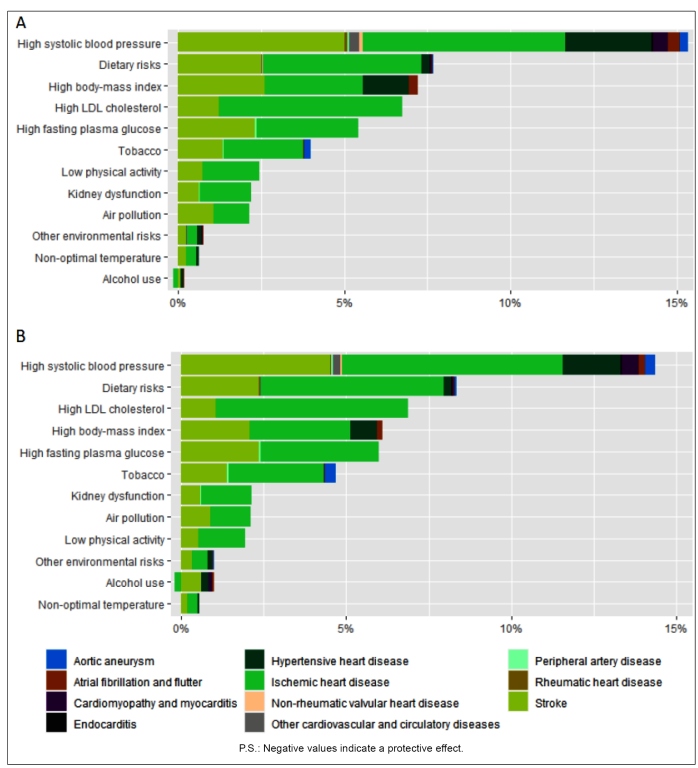
Percent of cardiovascular deaths by cause attributable to selected risk factors in 2019, in Brazil, for females **(A)** and males **(B)**.

Figure 3 shows the percentage of the total number of deaths from CVDs attributable to cardiovascular RF in Brazil. For both sexes, high SBP was the main RF, responsible for 6.3% of IHD deaths and 5.2% of stroke deaths for women, and 6.8% and 4.6% of IHD and stroke deaths for men, respectively. Dietary risks were the second most important RF, contributing to 5.0% and 5.7% of the deaths due to IHD and 2.6% and 2.4% of the deaths due to stroke, for females and males, respectively. Metabolic RF (LDL-cholesterol, high glycemia, high BMI, and kidney dysfunction), for both sexes, contributed mostly to mortality by IHD and stroke. It is notable that high BMI has a relevant contribution to mortality due to hypertensive cardiac disease. Tobacco, besides contributing to IHD and stroke, is responsible for 0.2% and 0.4% of the deaths by aorta aneurysm in men and women, respectively. 

**FIGURE 4:**
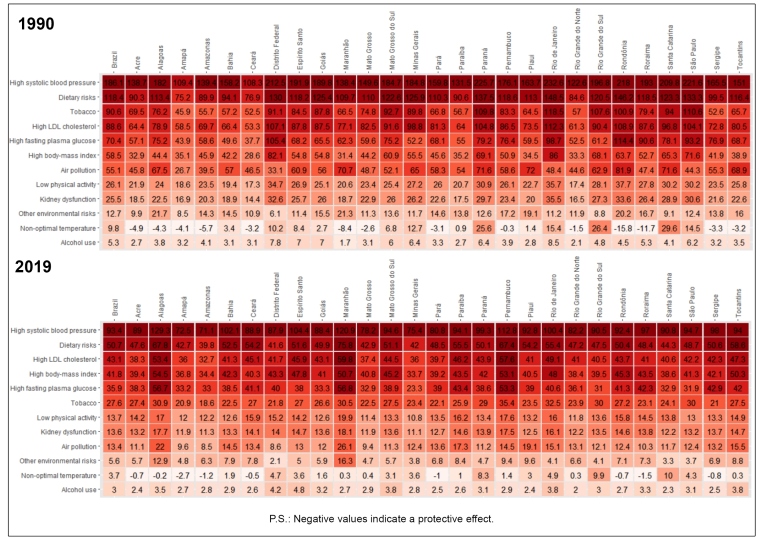
Heat map with age-standardized cardiovascular mortality rates (per 100,000 inhabitants) attributable to risk factors in 1990 and 2019 in Brazil and Federated Units.

When analyzing the mortality rate from CVDs attributable to RF by Brazilian state (Figure 4), it was noted that high SBP ranked number one in every state in 1990 and 2019. It is also relevant that, in 1990, CVDs mortality rates per 100,000 inhabitants attributable to SBP was the highest in Rio de Janeiro (232.6), Paraná (225.7), São Paulo (221.6), Rondônia (218.0), and the Federal District (212.5), whereas in 2019, that rate was the highest in Alagoas (129.3), Maranhão (120.9), Pernambuco (112.8), Espírito Santo (104.4), and Bahia (102.1). Tobacco, fasting plasma glucose, LDL-cholesterol, high BMI, and dietary risks were among the five most important risk factors in the majority of the states in 1990 and 2019. In 1990, the highest mortality rates across RF were concentrated in the Federal District and the states of São Paulo, Paraná, and Santa Catarina, and in 2019, these were found in the states of Alagoas, Maranhão, and Pernambuco.  Supplementary Material Figure 3 shows similar patterns for the DALY rates from CVDs attributable to RF for the states. 

**FIGURE 5:**
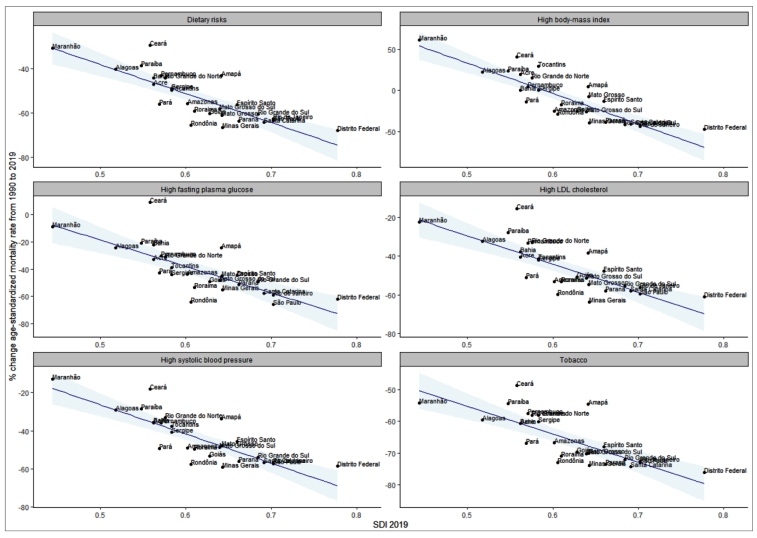
Relation between sociodemographic index and relative change in age-standardized mortality rates due to cardiovascular diseases attributable to selected risk factors from 1990 to 2019, in Brazilian Federated Units.

Figure 5 shows the relative change in mortality rates due to CVDs attributable to selected RF by SDI. CVDs deaths attributable to dietary risks, to high glycemia, high LDL-cholesterol, SBP, and tobacco diminished in every state, but these changes were more expressive in states with high SDI. For deaths attributable to high BMI, there was an increase in mortality rates in the least developed states, while the most developed states presented a reduction.

## DISCUSSION

This study shows that there has been progress in reducing exposure to RF for cardiovascular diseases since 1990, which is reflected in the consistent decrease in the age-standardized CVDs mortality and DALY rates attributable to the evaluated RF. However, with the exception of tobacco and environmental factors, there was a stability or increase in the SEV and of the crude mortality rates and DALYs attributable to these RF, especially in the case of metabolic risk factors, such as high glycemia and high BMI. Moreover, the decrease in the mortality due to CVDs attributable to RF was unequal among Brazilian states and was more significant in the states with better sociodemographic conditions. 

CVDss are still the main cause of death in Brazil^3^. The success in controlling risk factors, such as high SBP and smoking, is commendable; however, the increase in metabolic RF is a reason for concern and is consistent with previously published data^20^ ^-^ ^22^, especially considering that the RF which increased the most was high BMI. Studies show that obesity, even when it is not accompanied by metabolic alterations, increases the risk of CVDs in individuals^23^. Moreover, obesity is also an intermediate RF in the chain of metabolic changes, preceding SBP, diabetes mellitus (DM), and dyslipidemia; besides also compromising the control of these conditions, thereby increasing the risks of the individual^23^. SBP and DM are, in turn, the main determining factors for chronic kidney disease (CKD)^24^, another RF for CVDs that also has been increasing in Brazil. Therefore, unless this trend changes, we can foresee an increase in the other metabolic RF and of CKD in the coming years. 

To change that trend, health policies aimed at promoting healthy behavior are essential in order to reduce dietary risks and the lack of physical activity, which are determining factors for obesity and for metabolic RF^1^ ^,^ ^5^ ^,^ ^25^ ^,^ ^26^. Such policies require an integrated approach, ranging from health professionals encouraging healthy habits customized to specific populations, as well as increased taxes on unhealthy foods (like ultra processed foods), tobacco, and alcohol. Furthermore, investments in urban planning are also needed so as to create conditions for the practice of physical activities for all age groups^5^ ^,^ ^25^ ^,^ ^27^ ^-^ ^30^. Moreover, controlling the intermediate RF in the causation chain of CVDs, such as SBP and DM, is also important in order to reduce the burden of disease attributable to these factors. Investments in primary care and in the renewal of policies of access to medication, improvements in public health campaigns, the adoption of innovative techniques, and the implementation of digital technology are also strategies to reduce the burden of the CVDs in Brazil.

In fact, following the Brazilian “Plan of Strategic Actions to Confront NCD, 2020-2021” (*Plano de Ações Estratégicas para o Enfrentamento das DCNT, 2020-2021*”, in Portuguese), successful initiatives have been implemented in the country aiming at preventing and controlling NCD. From these, we could highlight: the implementation of community gyms^31^, that increased physical activity of the Brazilian population^32^; the enforcement of anti-smoking laws^33^; and the release of the “Brazilian Food Guide” in 2014^34^, along with partnerships with the food sector to reduce sodium of processed foods^35^. However, some of the initiatives described may negatively suffer the effect of the economic crisis and implementation of austerity measures in the country.

In terms of tackling smoking, new challenges lie ahead. The number of smokers has remained stable in recent years; smoking has increased among young people of 18 to 24 years of age, and the consumption of electronic cigarettes has increased, which, although illegal since 2009, are sold illegally in the country^8^. These are examples which show that the anti-smoking policies require strengthening and renewal. In addition, since the beginning of the COVID-19 pandemic, exposure to smoking has increased, as well as other unhealthy habits, like sedentarism^36^, and the control of the RF, like SBP and DM, has worsened, with a possible negative impact in the near future. 

Concerning heterogeneity in the decline of mortality by CVDs attributable to risk factors among the Brazilian states, there is a need for specific approaches aimed at controlling the impact of metabolic RF, geared toward the states with lower socioeconomic development so as to reduce these inequalities. Such approaches must consider the local culture and local dietary habits, and must use a language that is accessible to these specific populations. Quality planning is needed to deal with a possible increase in acute and chronic CVDs in the near future in order to avoid the continuation of the inequality cycle. Lastly, but of no less importance, an effort to reduce inequalities is essential, with interventions in the social determinants of health in an attempt to reduce non-communicable diseases (NCDs)^30^ ^,^ ^37^ ^,^ ^38^. 

This study has limitations. The limitations of the GBD models related to the causes of death have been discussed previously^16^ ^,^ ^17^. Concerning the evaluation of risk factors, one of the premises of the GBD study is to assume that the RRs are distributed evenly in every country, for determined age groups and sex^6^, and, as such, caution is required to properly interpret the data. Using SEV instead of prevalence as a measure of exposure to risk is an option which makes it difficult to compare data with primary studies. Moreover, more distant risk factors such as social determinants of health, were not evaluated in the GBD, and it is well-known that they can have an impact on the prevalence of intermediate risk factors even before affecting the estimates of attributable mortality^37^ ^,^ ^38^. Another aspect that must also be evaluated is the effect of the presence of many RF simultaneously, since it is well-known that the risk of these aggregated factors for the CVDs is greater than the sum of the risks of each individual risk factor^1^ ^,^ ^39^. Finally, even though the GBD methodology adjusts different definitions of the RF into a standardized definition, these adjustments may not be enough, thereby causing discrepancies^6^ ^,^ ^12^.

However, the results of the GBD study are important, since they provide a comprehensive and detailed evaluation of the trends of risk factors and their impact on CVDs in Brazil, contributing to identify knowledge gaps. These results can also help to guide public policies by showing, for instance, the need to address the problem of obesity in states with lower socioeconomic development. It is worth mentioning that Brazil has an important contribution to the GBD estimates through studies and inquiries on risk factors, such as the National Health Survey, telephone surveys, longitudinal studies^8^ ^-^ ^11^, as well as a network of collaborators who work with the IHME in the discussion and revision of estimates for Brazil^40^.

In conclusion, twelve RF are responsible for more than 80% of the CVDs burden in Brazil, and even though there was a decline in the age-standardized mortality rates attributable to such factors, those rates remain high and have different magnitudes among Brazilian states, affecting mainly the states with worse socioeconomic conditions. Among the RF for CVDs, the growing impact of the metabolic RF - especially obesity - stands out. This data, combined with the growing number of deaths attributable to cardiovascular risk factors, due to the growth and aging of the Brazilian population, demand that policies to reduce those risk factors be prioritized and renewed, focused on the promotion of health, combined with more investments in prevention, so as to reduce the burden of CVDs in Brazil. In so doing, the country will take steps towards reaching one of the Sustainable Development Goals proposed by the World Health Organization for 2030^41^: reduce by one third the premature mortality caused by NCDs through prevention and treatment, as well as reduce inequalities.
