# Supplementary material for: Burden of Cardiovascular diseases attributable to risk factors in Brazil: data from the "Global Burden of Disease 2019" study
Source: Rev Soc Bras Med Trop. 2022 Jan 28;55(Suppl 1):e0263-2021. doi: 10.1590/0037-8682-0263-2021 (PMC9009428; doi:10.1590/0037-8682-0263-2021)
Supplement: Supplementary file 1 [file 1678-9849-rsbmt-55-s01-e0263-2021-supp1.pdf]

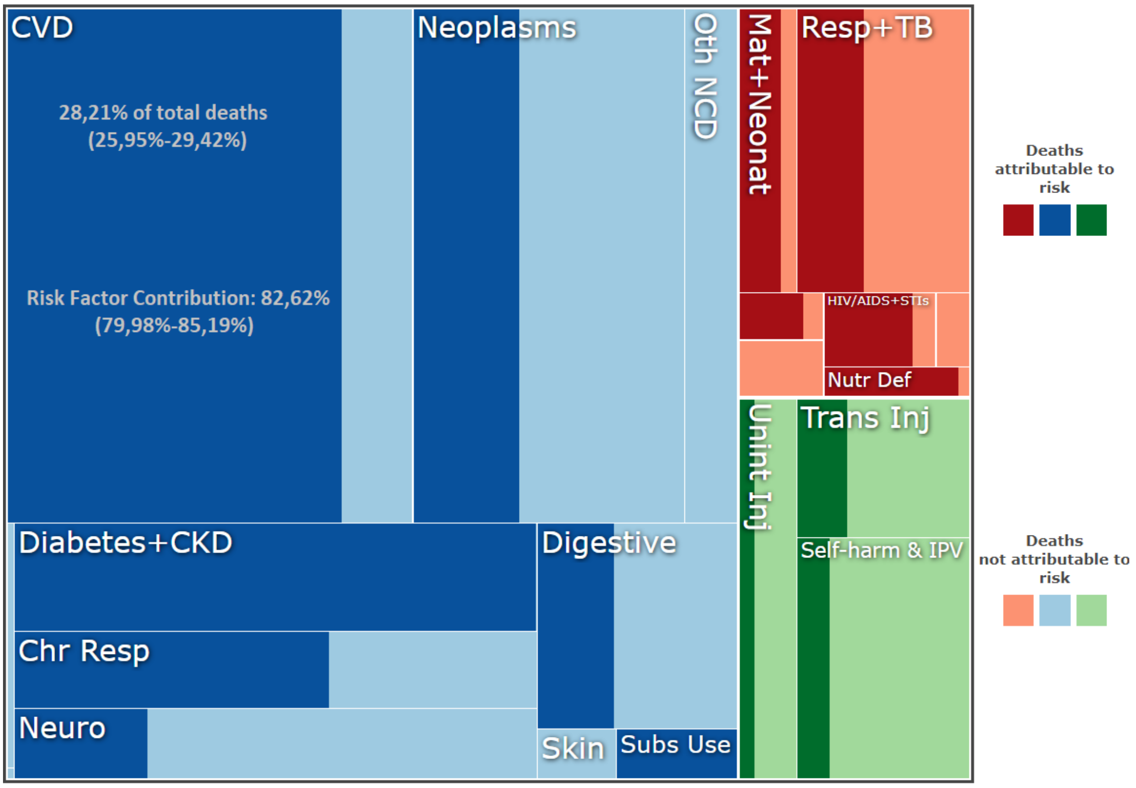

Supplemental Figure 1. Proportional deaths due to "Global Burden of Disease" study's level 2 causes add their risk factor attribution. Brazil, 2019.
