# Supplementary material for: Burden of Cardiovascular diseases attributable to risk factors in Brazil: data from the "Global Burden of Disease 2019" study
Source: Rev Soc Bras Med Trop. 2022 Jan 28;55(Suppl 1):e0263-2021. doi: 10.1590/0037-8682-0263-2021 (PMC9009428; doi:10.1590/0037-8682-0263-2021)
Supplement: Supplementary file 2 [file 1678-9849-rsbmt-55-s01-e0263-2021-supp2.pdf]

Supplemental Table 1. Cardiovascular risk factors summary exposure values and 95% uncertainty intervals, in Brazil, for males and females, in 1990 and 2019, and percent change.

Risk Factor	Female									Male									Both								
	1990			2019			PC %			1990			2019			PC %			1990			2019			PC %		
	Val	Interval		Val	Interval		Val	Interval		Val	Interval		Val	Interval		Val	Interval		Val	Interval		Val	Interval		Val	Interval	
Air pollution	23.3	14.7	33.7	9.3	5.8	13.4	-60.0	-73.6	-40.2	23.4	14.4	33.9	9.4	5.9	13.4	-59.9	-73.4	-39.6	23.3	14.7	33.9	9.3	5.9	13.3	-59.9	-73.6	-40.0
Alcohol use	3.3	2.1	4.9	4.9	3.2	7.1	48.5	21.7	82.9	10.0	7.1	13.5	13.8	9.9	18.4	37.9	20.8	61.3	6.6	4.6	9.0	9.3	6.4	12.5	41.0	24.2	63.4
Dietary risks	36.0	23.2	52.2	35.6	24.6	50.1	-1.0	-13.0	12.8	43.5	25.0	59.6	43.3	25.4	59.2	-0.5	-16.8	20.4	39.6	24.7	55.2	39.3	25.4	53.7	-0.9	-12.1	13.5
High body-mass index	16.2	11.4	22.7	31.9	26.0	40.0	96.6	65.1	145.7	13.4	9.1	19.5	30.4	24.0	38.9	126.9	87.7	197.4	14.9	10.4	20.5	31.2	25.5	39.5	110.2	78.6	161.7
High fasting plasma glucose	9.6	8.4	10.8	10.7	9.5	12.0	11.7	3.6	19.7	10.3	9.0	11.6	12.3	10.8	13.9	19.0	11.2	27.5	9.9	8.7	11.1	11.4	10.1	12.8	15.1	9.3	21.2
High LDL cholesterol	41.2	37.8	44.7	46.0	42.6	49.4	11.8	5.8	18.7	39.3	35.8	42.9	43.9	40.3	47.6	11.6	4.8	19.8	40.3	37.0	43.7	45.1	41.9	48.3	11.8	6.9	17.2
High systolic blood pressure	26.1	23.1	29.4	24.0	21.7	26.3	-8.1	-19.3	4.3	28.1	25.4	30.6	30.8	28.4	33.2	9.6	1.0	19.3	27.1	24.8	29.6	27.3	25.2	29.3	0.7	-6.9	8.3
Kidney dysfunction	19.0	13.1	26.4	21.4	15.2	29.0	12.4	8.7	17.8	18.7	12.8	25.9	20.9	14.8	28.2	11.7	7.9	17.5	18.9	13.0	26.2	21.2	15.1	28.6	12.0	8.4	17.2
Low physical activity	12.6	8.0	19.1	13.2	8.6	19.2	4.7	-2.7	17.6	11.4	6.3	19.5	11.8	6.7	19.9	3.1	-8.2	20.3	12.0	7.2	19.5	12.5	7.7	19.2	3.9	-5.0	17.5
Non-optimal temperature	24.0	18.1	31.3	23.2	17.5	30.6	-3.6	-8.0	0.6	24.0	18.2	31.3	23.2	17.5	30.6	-3.7	-8.1	0.7	24.0	18.1	31.3	23.2	17.5	30.6	-3.6	-8.0	0.6
Other environmental risks	45.4	29.2	57.9	27.8	15.9	41.6	-38.7	-48.3	-27.9	56.7	45.0	66.0	35.4	23.9	48.4	-37.6	-47.4	-26.0	50.9	36.9	61.8	31.4	19.7	44.7	-38.3	-47.9	-27.1
Tobacco	30.2	28.0	32.5	20.7	18.9	22.6	-31.4	-36.8	-25.1	36.4	34.1	38.9	23.5	21.1	25.9	-35.5	-41.7	-29.0	33.2	31.1	35.4	22.0	20.4	23.7	-33.7	-38.2	-28.9

Abbreviations: LDL: low-density lipoprotein; PC%: percent change; Val: value (central estimate).
