# Supplementary material for: Burden of Cardiovascular diseases attributable to risk factors in Brazil: data from the "Global Burden of Disease 2019" study
Source: Rev Soc Bras Med Trop. 2022 Jan 28;55(Suppl 1):e0263-2021. doi: 10.1590/0037-8682-0263-2021 (PMC9009428; doi:10.1590/0037-8682-0263-2021)
Supplement: Supplementary file 3 [file 1678-9849-rsbmt-55-s01-e0263-2021-supp3.pdf]

A

1990			2019		
**Rank**	**Rate**	**Risk Factor**	**Rank**	**Rate**	**Risk Factor**
1	3264	High systolic blood pressure	1	1552	High systolic blood pressure
2	1977	Dietary risks	2	924	High body-mass index
3	1721	Tobacco	3	831	Dietary risks
4	1462	High body-mass index	4	692	High LDL cholesterol
5	1426	High LDL cholesterol	5	524	Tobacco
6	1075	Air pollution	6	484	High fasting plasma glucose
7	1008	High fasting plasma glucose	7	262	Air pollution
8	405	Kidney dysfunction	8	204	Kidney dysfunction
9	387	Low physical activity	9	197	Low physical activity
10	180	Other environmental risks	10	69	Other environmental risks
11	160	Non-optimal temperature	11	55	Non-optimal temperature
12	25	Alcohol use	12	15	Alcohol use

B

1990			2019		
**Rank**	**Rate**	**Risk Factor**	**Rank**	**Rate**	**Risk Factor**
1	4825	High systolic blood pressure	1	2561	High systolic blood pressure
2	3374	Dietary risks	2	1546	Dietary risks
3	3008	Tobacco	3	1315	High body-mass index
4	2496	High LDL cholesterol	4	1311	High LDL cholesterol
5	1768	High body-mass index	5	972	Tobacco
6	1618	Air pollution	6	906	High fasting plasma glucose
7	1593	High fasting plasma glucose	7	421	Air pollution
8	626	Kidney dysfunction	8	352	Kidney dysfunction
9	483	Low physical activity	9	276	Low physical activity
10	388	Other environmental risks	10	183	Alcohol use
11	369	Alcohol use	11	150	Other environmental risks
12	234	Non-optimal temperature	12	88	Non-optimal temperature

Metabolic risks   Environmental/occupational risks   Behavorial risks

Supplemental Figure 2. Ranking of age-standardized DALY rates (per 100,000 inhabitants) for cardiovascular diseases attributable to risk factors in 1990 and 2019 in Brazil, for females and males.
