# Supplementary material for: Burden of Cardiovascular diseases attributable to risk factors in Brazil: data from the "Global Burden of Disease 2019" study
Source: Rev Soc Bras Med Trop. 2022 Jan 28;55(Suppl 1):e0263-2021. doi: 10.1590/0037-8682-0263-2021 (PMC9009428; doi:10.1590/0037-8682-0263-2021)
Supplement: Supplementary file 4 [file 1678-9849-rsbmt-55-s01-e0263-2021-supp4.pdf]

Supplemental Table 2. Crude and age-standardized mortality rates due to cardiovascular diseases attributable to risk factors in 1990 and 2019, and percent change (%). Brazil, 1990 and 2019.

	All ages									Ages-standardized								
	1990			2019			PC %			1990			2019			PC %		
RiskFactor	val	Interval		val	Interval		val	Interval		val	Interval		val	Interval		val	Interval	
Air pollution	31.0	22.7	40.1	14.5	10.6	18.9	-53.3	-67.0	-34.5	55.1	40.4	71.9	13.4	9.8	17.6	-75.6	-82.9	-66.0
Alcohol use	3.8	2.1	5.6	3.3	1.6	5.2	-11.8	-40.8	25.4	5.3	2.4	8.5	3.0	1.4	4.8	-43.5	-62.5	-11.6
Dietary risks	62.8	50.5	77.9	53.9	41.8	69.8	-14.1	-21.8	-7.0	118.4	94.6	148.9	50.7	39.2	65.7	-57.2	-60.9	-53.9
High body-mass index	35.4	20.2	53.0	45.5	30.8	61.8	28.5	11.1	63.2	58.5	32.7	89.7	41.8	28.1	56.8	-28.5	-38.8	-8.6
High fasting plasma glucose	34.1	24.0	49.1	37.3	25.8	54.5	9.2	-2.2	21.4	70.4	47.4	106.1	35.9	24.5	53.0	-49.0	-53.4	-43.9
High LDL cholesterol	45.9	37.0	56.3	45.9	36.0	58.2	-0.1	-7.4	6.7	88.6	67.8	114.8	43.1	33.4	55.9	-51.3	-53.8	-48.6
High systolic blood pressure	97.2	87.0	106.9	98.6	85.4	109.7	1.4	-5.1	7.3	186.1	163.8	206.7	93.4	80.2	104.2	-49.8	-52.5	-47.1
Kidney dysfunction	12.9	10.3	15.5	14.3	11.4	17.4	10.6	2.7	18.2	25.5	19.9	31.3	13.6	10.8	16.7	-46.5	-49.6	-43.4
Low physical activity	11.5	5.3	19.2	14.0	7.7	21.4	21.1	5.4	55.1	26.1	12.6	41.4	13.7	7.6	20.8	-47.6	-53.6	-35.0
Non-optimal temperature	5.0	1.2	7.8	3.9	0.9	5.9	-23.1	-61.2	53.0	9.8	1.8	15.4	3.7	0.8	5.6	-62.3	-78.6	-19.0
Other environmental risks	6.7	3.2	10.3	5.8	2.7	9.3	-13.3	-22.5	-4.9	12.7	6.2	19.5	5.6	2.6	8.9	-56.1	-60.0	-52.8
Tobacco	53.6	50.5	56.8	30.3	28.0	32.5	-43.4	-47.9	-39.1	90.6	84.6	96.5	27.6	25.5	29.7	-69.5	-72.0	-67.1

Abbreviations: LDL: low-density lipoprotein; PC%: percent change; Val: value (central estimate).
