# Supplementary material for: Burden of Cardiovascular diseases attributable to risk factors in Brazil: data from the "Global Burden of Disease 2019" study
Source: Rev Soc Bras Med Trop. 2022 Jan 28;55(Suppl 1):e0263-2021. doi: 10.1590/0037-8682-0263-2021 (PMC9009428; doi:10.1590/0037-8682-0263-2021)
Supplement: Supplementary file 5 [file 1678-9849-rsbmt-55-s01-e0263-2021-supp5.pdf]

1990

	Brazil	Acre	Alagoas	Amapá	Amazonas	Bahia	Ceará	Distrito Federal	Espírito Santo	Goiás	Maranhão	Mato Grosso
High systolic blood pressure	4011	2710	3900	2205	2707	3426	2345	3971	3914	4119	3277	3118
Dietary risks	2641	1866	2570	1556	1811	2153	1695	2533	2513	2793	2742	2372
Tobacco	2332	1640	1952	1140	1328	1512	1309	2146	2140	2318	1896	1886
High LDL cholesterol	1940	1293	1764	1179	1338	1490	1149	2004	1773	1892	1952	1773
High body-mass index	1612	884	1259	957	1183	1206	791	2031	1506	1526	950	1201
Air pollution	1334	1033	1628	620	888	1407	1101	729	1444	1384	1830	1160
High fasting plasma glucose	1280	942	1387	723	966	926	662	1726	1165	1165	1242	1036
Kidney dysfunction	511	346	467	315	371	390	286	566	496	520	420	446
Low physical activity	435	336	402	283	353	327	291	508	411	403	384	382
Other environmental risks	277	203	493	176	296	332	240	116	240	345	530	239
Non-optimal temperature	196	-86	-85	-73	-98	69	-60	175	157	53	-185	-49
Alcohol use	189	91	135	107	133	121	106	247	233	238	74	114

	Mato Grosso do Sul	Minas Gerais	Pará	Paraíba	Paraná	Pernambuco	Piauí	Rio de Janeiro	Rio Grande do Norte	Rio Grande do Sul	Rondônia	Roraima	Santa Catarina	São Paulo	Sergipe	Tocantins
High systolic blood pressure	3997	3919	3173	2833	4625	3795	3297	5205	2578	4126	4146	3641	4312	4616	3203	2851
Dietary risks	2687	2809	2263	2047	2892	2608	2388	3404	1825	2605	2833	2353	2563	2865	2001	2318
Tobacco	2344	2386	1636	1465	2705	2125	1641	3112	1404	2689	2298	1869	2319	2777	1303	1610
High LDL cholesterol	1966	2067	1621	1437	2147	1878	1531	2542	1312	1941	2061	1713	1961	2198	1403	1560
High body-mass index	1677	1573	1197	979	1807	1394	931	2415	915	1813	1575	1310	1660	1887	1094	996
Air pollution	1277	1614	1309	1314	1658	1403	1634	1232	1049	1489	1731	1045	1608	1069	1217	1493
High fasting plasma glucose	1319	961	1125	994	1354	1366	998	1889	924	1075	1851	1510	1316	1609	1308	1085
Kidney dysfunction	510	519	426	356	566	466	384	728	321	526	594	480	538	583	397	404
Low physical activity	410	435	396	361	485	431	347	615	282	457	568	415	481	478	346	375
Other environmental risks	295	261	304	308	267	378	404	247	257	201	392	331	184	253	283	316
Non-optimal temperature	131	248	-54	18	473	-5	25	312	-28	500	-270	-205	539	273	-56	-54
Alcohol use	203	245	119	97	206	141	100	292	81	173	142	166	147	216	115	120

2019

	Brazil	Acre	Alagoas	Amapá	Amazonas	Bahia	Ceará	Distrito Federal	Espírito Santo	Goiás	Maranhão	Mato Grosso
High systolic blood pressure	2019	1791	2829	1591	1459	2239	1896	1540	2173	1953	2559	1673
Dietary risks	1161	1035	1601	971	882	1244	1190	792	1166	1163	1705	991
High body-mass index	1109	1017	1488	995	898	1158	1053	971	1236	1089	1379	1069
High LDL cholesterol	981	807	1266	800	697	974	968	751	1018	1006	1341	852
Tobacco	731	686	817	550	469	605	681	518	708	714	809	596
High fasting plasma glucose	673	683	1135	623	608	757	733	632	698	633	1063	622
Air pollution	336	269	560	237	205	373	323	185	320	303	639	236
Kidney dysfunction	272	251	375	239	220	281	269	232	290	275	363	241
Low physical activity	233	227	305	201	199	219	260	213	240	219	326	196
Other environmental risks	105	104	263	92	120	159	144	31	92	113	313	88
Alcohol use	93	73	109	85	83	91	84	108	142	99	86	87
Non-optimal temperature	70	-13	-4	-52	-22	38	-10	73	70	31	4	7

	Mato Grosso do Sul	Minas Gerais	Pará	Paraíba	Paraná	Pernambuco	Piauí	Rio de Janeiro	Rio Grande do Norte	Rio Grande do Sul	Rondônia	Roraima	Santa Catarina	São Paulo	Sergipe	Tocantins
High systolic blood pressure	2073	1694	1746	2062	2041	2531	1943	2302	1790	1895	1943	1831	1822	2031	2087	1923
Dietary risks	1171	993	1108	1288	1100	1565	1225	1313	1099	1033	1122	995	950	1117	1156	1300
High body-mass index	1188	934	1050	1165	1064	1418	1077	1306	1025	1003	1168	1035	947	1087	1120	1283
High LDL cholesterol	1016	827	896	1071	960	1335	930	1167	961	873	966	825	861	964	951	1045
Tobacco	728	644	575	690	747	936	624	875	626	781	697	578	629	799	565	717
High fasting plasma glucose	726	450	740	837	699	1017	725	790	694	559	762	737	577	586	821	766
Air pollution	283	318	336	441	269	366	475	390	335	290	303	239	275	312	327	380
Kidney dysfunction	272	226	256	297	269	353	251	330	246	258	286	250	226	259	277	288
Low physical activity	228	184	228	290	218	310	214	290	207	220	272	218	221	220	227	244
Other environmental risks	107	74	133	164	84	184	181	78	127	80	132	124	57	65	134	160
Alcohol use	114	92	78	85	87	96	75	117	65	89	80	93	66	92	80	110
Non-optimal temperature	59	72	-20	19	151	27	56	99	4	180	-13	-26	177	83	-15	5

P.S.: Negative values indicate a protective effect.

Supplemental Figure 3. Heat map with age-standardized cardiovascular DALY rates (per 100,000 inhabitants) attributable to risk factors in 1990 and 2019 in Brazil and Federated Units.
